# Aplasia cutis congenita of the trunk in a newborn: a rare case report

**DOI:** 10.11604/pamj.2024.48.52.43784

**Published:** 2024-06-09

**Authors:** El Mouloua Ahmed, Aballa Najoua, Foura Salma, Kamili Elouafi Elaouni, Fouraiji Karima, Oulad Saiad Mohamed

**Affiliations:** 1 General Pediatric Surgery unit, Mother and Child Department, Mohamed VI Teaching Hospital, Cadi Ayyad University, Marrakech, Morocco

**Keywords:** Skin defect, aplasia cutis congenita, trunk, newborn, case report

## Abstract

Aplasia cutis congenita (ACC) is a rare congenital disorder defined as a congenital skin defect, characterized by the absence of all skin layers at birth. The most frequent presentation is a small erythematous ulcerated or scar-like alopecic ectodermal lesion on the scalp vertex. However, extensive cutis aplasia of the trunk is extremely uncommon. Clinical and radiological evaluation defined the appropriate treatment. We herein report a rare case of a large aplasia cutis congenita of the trunk occurring in a male newborn managed with sulfadiazine silver 1% dressing, complete healing was achieved in about a month. The report highlights that conservative treatment is a highly effective and practical option for managing non-scalp extensive ACC.

## Introduction

Aplasia cutis congenita (ACC) is a rare congenital disorder involving variable skin layers. It is a rare congenital abnormality that mostly appears as solitary lesions involving the midline over the skull vertex, but other sites, such as the trunk or limbs can be involved [1,2]. The etiology of ACC is still unclear despite many pathogenesis theories. It appears to be multifactorial [3]. The management of non-scalp ACC is still controversial. Herein, we present a case of successful conservative treatment of a male newborn with a large AAC involving the trunk.

## Patient and observation

**Patient information:** a full-term baby boy was transferred to the general pediatric surgery unit, for the absence of skin in large trunk areas. He was delivered normally with a birth weight of 3200 g. He was the 4^th^ child of non-consanguineous healthy parents. There was no history of drug intake by the mother during pregnancy. Weekly ultrasounds of the fetus were not performed during pregnancy. Also, no history of infectious disease or chronic illness was noted. There was no such case in the family.

**Figure 1:**
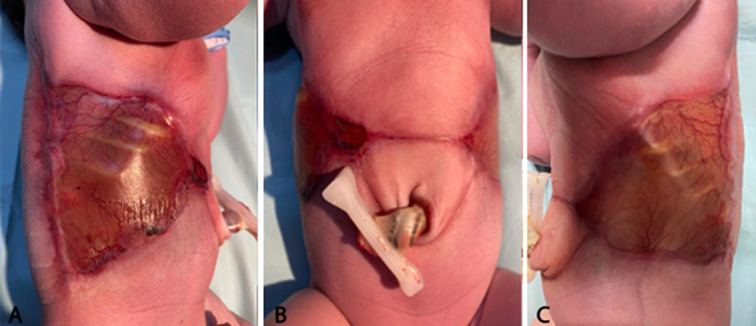
on day one: the baby showing a large aplasia cutis congenita on both sides of the trunk linked to a narrow band, passing just below the umbilicus; A) the right side of the trunk; B) the ventral side; C) the left side of the trunk

**Clinical findings:** the physical exam showed a triangular, symmetric bilateral skin defect of the trunk. The concerned areas were covered by a transparent membrane. The underlying blood vessels, the ribs, and the small bowel appear through the transparent membranous (Figure 1). There was no bone defect. These two triangles were linked to a narrow band, passing just below the umbilicus.

**Diagnostic assessment:** chest, abdomen, and spinal radiographs were normal, and the kidney ultrasound and the echocardiography didn´t show any malformations. Blood tests and laboratory tests examining kidney and liver function were normal.

**Diagnosis:** through clinical findings, the patient was diagnosed with ACC. We didn´t realize any skin biopsy.

**Therapeutic interventions:** the patient was managed conservatively. After gentle cleansing of the denuded area with saline, sulfadiazine silver 1% dressing and bandaging were applied, once a day for one week, then every 2 days for 3 weeks. unnecessary handling of the baby was avoided to prevent abrasions on the delicate and thin membrane.

**Figure 2:**
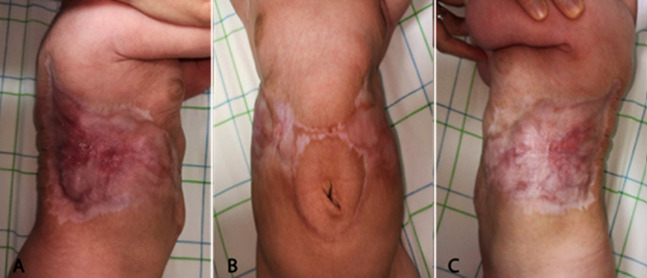
A, B, C) aspect after 1 month, complete healing of the skin defect with mature scar tissue and slight abnormal hypopigmentation

**Follow-up and outcome of interventions:** complete healing was obtained in about one month, with mature scar tissue (Figure 2), and no surgical intervention was required.

**Patient perspective:** the parents were unequivocally satisfied with the results and expressed their utmost gratitude for the exceptional care and treatment provided.

**Informed consent:** the parents of the patient provided informed consent.

## Discussion

Aplasia cutis congenita is a rare congenital disorder. Its incidence is estimated between 0.5 and 3 per 10,000 births [4]. Aplasia cutis congenita is characterized by the complete or partial skin defect at birth of one or several areas, less commonly periosteum bone can be involved. While frequently an isolated disorder, it may be associated with other anomalies such as Adams-Oliver syndrome, Fetus papyraceous, or epidermolysis bullosa [4]. Most of the cases affect the scalp (86%) as single lesions [2,4]. The non-scalp lesions are usually bilateral and symmetrical, mainly involving the trunk, as in our case, or the extremities [5]. The etiology behind the issue is still not well defined, because of the variety of presentation and severity. Still, many hypotheses have been suggested, including vascular, genetic, traumatic, pharmacological, or an anomaly in the neural tube closure process [4]. No etiological factors were encountered in this case, and the parents were not closely related.

Frieden IJ have distinguished 9 subtypes of ACC, distributed according to the affected areas, the associated anomalies, and the possible mode of inheritance, they are described as follows: (1) ACC of the scalp without multiple abnormalities, (2) ACC of the scalp with associated limb abnormalities, (3) ACC of the scalp with associated epidermal and organoid nevi, (4) ACC overlying embryologic malformations, (5) ACC associated with fetus papyraceus or placental infarcts, (6) ACC associated with epidermolysis bullosa, (7) ACC localized to the extremities without blistering and without associated abnormalities, (8) ACC caused by specific teratogens, (9) ACC associated with malformation syndromes [6]. Our patient did not match any of the subtypes in Frieden's classification system. Therefore, we suggest adding a new group to the classification system to include this rare form of ACC. Aplasia cutis congenita (ACC) is primarily a clinical diagnosis. However, several differential diagnoses should be considered as epidermolysis bullosa without ACC, post-traumatic skin loss, burns, neonatal gangrene, necrotizing fasciitis, or congenital varicella [4].

The management of ACC is dependent on the surface of the affected area, localization, and associated malformations, it aims to avoid friable tissue perforation and infection. Conservative management has been proven to be highly successful in treating large ACC, as it allows gradual epithelialization of skin and atrophic scar over several weeks, thus avoiding the potential operative risk to a newborn [3,5,7]. Surgical treatments may be required either urgently or secondarily, particularly in cases involving the scalp. It includes split-thickness or full-thickness skin grafts and tissue expansion. Simman R *et al*. have described a technique of coverage of large skin absence using allogeneic dermis and cultured epithelial autografts in newborns with good outcomes [8], but their use is restricted to centers having tissue culture laboratories. The prognosis of ACC depends on the nature and severity of associated conditions or malformations, although it is usually excellent [7].

## Conclusion

We think that conservative management is the gold standard for treating ACC, it is a simple and effective treatment approach even for large non-scalp ACCs, and it thus avoids the operative risks in newborns. It should be initiated promptly after birth to prevent perforation and infection.

## References

[1] Mesrati H, Amouri M, Chaaben H, Masmoudi A, Boudaya S, Turki H (2015). Aplasia cutis congenita: report of 22 cases. Int J Dermatol.

[2] Demmel U (1975). Clinical aspects of congenital skin defects. Eur J Pediatr.

[3] Abulezz TA, Shalkamy MA (2009). Aplasia cutis congenita: Two cases of non-scalp lesions. Indian J Plast Surg.

[4] Belkhou A, François C, Bennis Y, Duquennoy-Martinot V, Guerreschi P (2016). Aplasia cutis congenita: mise au point et prise en charge. Ann Chir Plast Esthet.

[5] Taifour Suliman M, Quazi A (2004). Aplasia cutis congenita of the trunk in a Saudi newborn. Br J Plast Surg.

[6] Frieden IJ (1986). Aplasia cutis congenita: A clinical review and proposal for classification. J Am Acad Dermatol.

[7] Higgins C, Price A, Craig S (2022). Aplasia cutis congenita. BMJ Case Rep.

[8] Simman R, Priebe CJ, Simon M (2000). Reconstruction of aplasia cutis congenita of the trunk in a newborn infant using acellular allogenic dermal graft and cultured epithelial autografts. Ann Plast Surg.

